# pedQTNet: A Deep Learning Approach to Estimate Corrected QT Intervals from Multi-Lead Conventional ECG Waveforms in Pediatric Patients

**DOI:** 10.1007/s10916-026-02386-1

**Published:** 2026-06-03

**Authors:** Victor M. Ruiz, Ivor B. Asztalos, Luiz E. V. Silva, Lingyun Shi, V. Ramesh Iyer, Dustin Nash, Victoria L. Vetter, Fuchiang Rich Tsui

**Affiliations:** 1 Tsui Laboratory, Department of Biomedical and Health Informatics, Children’s Hospital of Philadelphia, Philadelphia, PA USA; 2 Division of Cardiology, Children’s Hospital of Philadelphia, Philadelphia, PA USA; 3 Division of Cardiology, Children’s Hospital of Colorado, Aurora, CO USA; 4 Department of Anesthesiology and Critical Care, University of Pennsylvania, Philadelphia, PA USA; 5 Department of Biomedical, Epidemiology, and Informatics, University of Pennsylvania, Philadelphia, PA USA; 6 Department of Anesthesiology and Critical Care Medicine, Children’s Hospital of Philadelphia, Philadelphia, PA USA; 7 Roberts Center for Pediatric Research, Children’s Hospital of Philadelphia, Philadelphia, PA USA

**Keywords:** QTc, Machine learning, Pediatric, Children, Cardiology, Electrocardiogram (ECG)

## Abstract

Long QT syndrome (LQTS) is a primary risk factor for ventricular arrhythmias and sudden cardiac death in children. Accurate corrected QT intervals (QTc) measurement is imperative but challenging for non-heart-rhythm specialists, especially in children. We developed and evaluated pedQTNet, a deep neural network model for estimating QTc and detecting LQTS in pediatric patients. We analyzed a cohort of 37,992 patients aged 0–18 years with 65,370 ECGs annotated by pediatric electrophysiologists (PEPs) between 2010 and 2020. Using PEP-annotated QTc measurements as ground truth, pedQTNet was trained and calibrated on raw ECG waveforms to optimize QTc estimation and LQTS classification. Performance was compared to GE Healthcare’s Marquette 12SL algorithm, and to PEPs in cross-validation, as well as an additional prospective set of 200 ECGs. In 10-fold cross-validation, pedQTNet estimated QTc’s with a mean absolute error (MAE) of 18.8 ms (95% CI: 18.4–19.2) and predicted LQTS at 470 ms with 85% sensitivity (83%-87%), 87% specificity (87%–88%), positive likelihood ratio (PLR) of 6.7 (6.5–7.0), and negative likelihood ratio (NLR) of 0.17 (0.15–0.19), outperforming Marquette 12SL. In the prospective set, pedQTNet had higher sensitivity than PEPs (100% [69%–100%] vs. 71% [53%–85%], *P* < 0.05), and a lower but not statistically significant NLR (0.00 [0.00–0.70] vs. 0.30 [0.18–0.50], *P* = 0.2). PedQTNet demonstrated high QTc estimation accuracy and reliable LQTS detection, outperforming a commercial tool and on par with expert interpretation. Its strong performance supports its clinical use for scalable, automated pediatric ECG screening and LQTS risk assessment, offering a practical tool for enhancing pediatric cardiac care.

**Supplementary Information:**

The online version contains supplementary material available at 10.1007/s10916-026-02386-1.

## Introduction

Long QT syndrome (LQTS) either congenital or acquired (e.g., drug-induced) is a primary risk factor for developing ventricular arrhythmias, specifically torsades de pointes (TdP), and sudden cardiac death (SCD) in children. [1] The prevalence of congenital LQTS is estimated to be between 1 in 2000 and 1 in 2,500 children. [2] While the prevalence of acquired LQTS in children is not known with precision, it is estimated to be higher than congenital LQTS. Importantly, acquired LQTS is especially prevalent in certain high-risk populations, e.g., 9% in pediatric oncology inpatients. [3] The morbidity and mortality attributed to congenital LQTS are significant, with 6% and 19% of individuals experiencing cardiac arrests by 14 and 40 years of age, respectively. [4, 5]

The hallmark of LQTS is a prolongation of heart-rate corrected QT intervals (QTc) on the surface electrocardiogram (ECG). While detection and management of LQTS can effectively minimize risk of cardiac arrest and death, early detection is challenging, making accurate and reliable measurement of the QTc imperative. Unfortunately, measurement of the QTc is not always straightforward and can be problematic for many non-heart-rythm, especially in the pediatric setting where ECG interpretation is less common. Automated computerized QTc measurement partially addresses this issue, but different algorithms have shown low levels of agreement with manual QTc measurement by physicians for both adult and pediatric ECGs. [6–9]

With the emergence of artificial intelligence in medicine, computer-aided diagnosis and prognosis of diverse medical conditions have become feasible. [10] Deep neural networks (DNNs) are particularly compelling in healthcare applications due to their ability to recognize complex data relationships without the need for feature engineering or elaborate data preprocessing. Recently, DNN models were successfully applied to characterize ECG patterns related to different conditions, including the classification of cardiac rhythms and electrical conduction disorders, [11–13] myocardial infarction, [14] and ventricular disfunction. [15]

Although recent studies have applied DNNs to QTc estimation and LQTS, their applicability to clinical pediatric practice is limited. [16, 17] To our knowledge, no currently published algorithms achieved the necessary performance to safely rule out LQTS. Furthermore, existing models have been trained and validated on exclusive or majority adult samples, thus limiting their generalizability to the pediatric population, in whom higher heart rate, larger RR-interval variability, and altered T-wave morphologies/axes impact models’ ability to measure the QTc.

In this study, we propose an innovative DNN model, pedQTNet, for QTc estimation and LQTS classification, either congenital or acquired, for clinical use in a large pediatric population using standard 15-lead pediatric surface ECGs. Additionally, we test two secondary hypotheses: (1) that similar performance can be achieved with a reduced set of leads; and (2) that our models’ performance is robust across ECGs with varying electrocardiographic phenomena that may affect the QTc. Our contribution lies in the development of a clinically-applicable model for pediatric QTc estimation and LQTS classification, which outperforms a current commercial algorithm and showcases the potential to, at least, match the proficiency of expert human readers.

## Methods

This study was approved by the Children’s Hospital of Philadelphia’s Institutional Review Board (IRB 20-018196) with a waiver of informed consent. The investigation conformed to the principles outlined in the Declaration of Helsinki and the guidelines of the Strengthening the Reporting of Observational Studies in Epidemiology (STROBE).

### Dataset

**Fig. 1:**
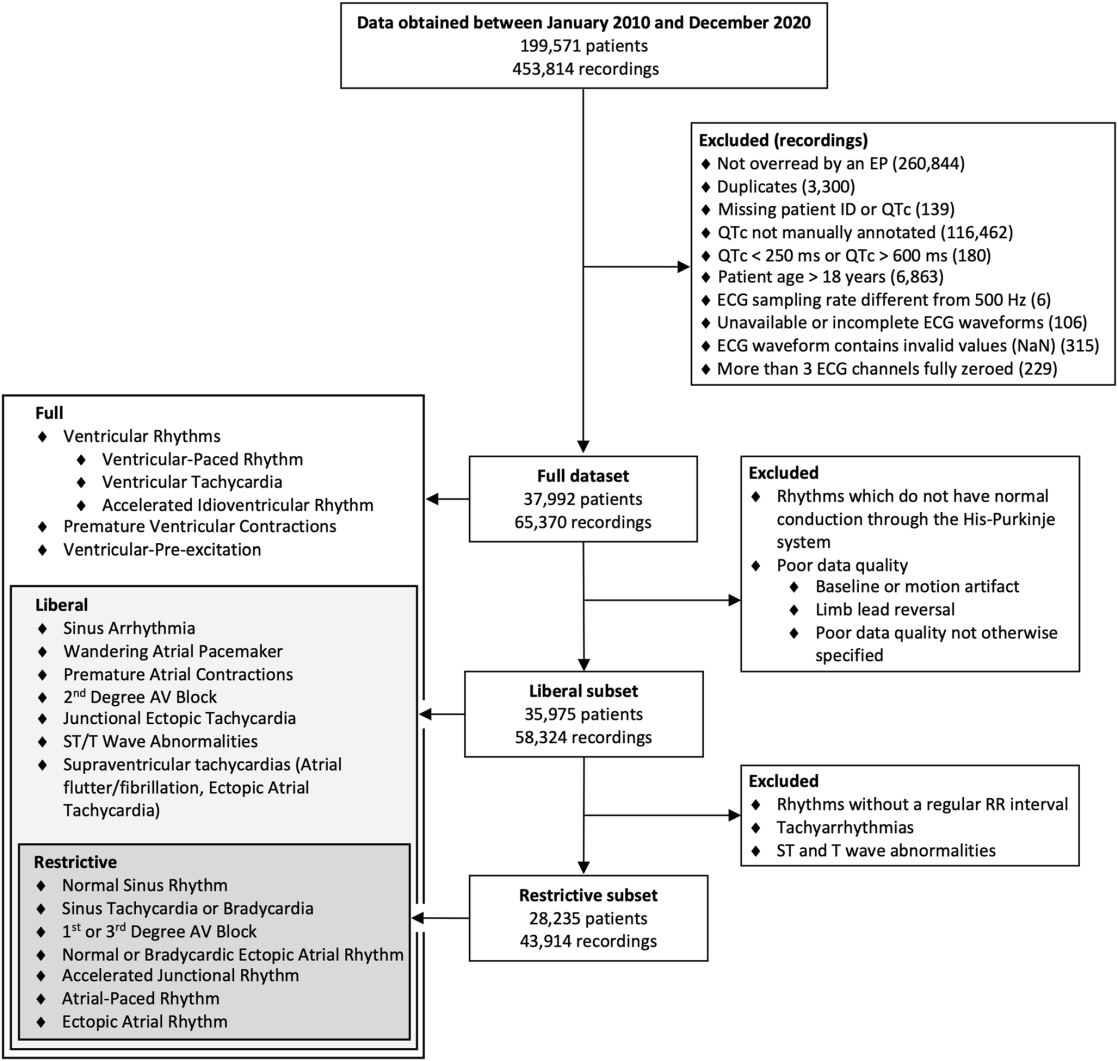
Flow diagram for patients and ECG recordings. Right boxes show the exclusion criteria while left boxes show the three groups evaluates. For details on the diagnosis labels adopted for the liberal and restrictive subsets, see Table S1 of supplementary material. EP: electrophysiologist; AV: atrio-ventricular

All resting, 10-second surface ECGs performed in inpatient and outpatient settings at a freestanding quaternary-care children’s hospital between January 2010 and December 2020 in children aged 0–18 years were included. ECGs were performed with standard 15-lead pediatric surface electrode configuration (leads I, II, III, aVR, aVL, aVF, V1-V7, V3r, V4r) sampled at 500 Hz on General Electric Healthcare (GE Healthcare, Chicago, IL, USA) devices (MAC VU360, MAC 5500, or MAC 5000). We applied several exclusion criteria (Fig. 1). We excluded leads III, aVR, aVL, and aVF, which can be derived from the remaining 2 limb leads, to minimize redundancy. We further excluded ECGs for which the QTc was not manually measured or overread by a pediatric electrophysiologist (PEP) using digital calipers in GE’s MUSE software. Finally, ECGs with QTc > 600 milliseconds (ms) or < 250 ms (considered spurious) and incomplete ECGs (< 10 s on 3 or more leads) were excluded. We grouped ECGs into three overlapping sets based on various electrocardiographic phenomena that may affect the QTc (Fig. 1). The first group (*full dataset*) included all recordings and is the main focus of our study findings. The second group (*liberal subset*) included all orthodromic rhythms irrespective of ST-segment or T-wave abnormalities as the clinical relevance of the QTc in ventricular and antidromic rhythms is limited. The third group (*restrictive subset*) included only stable atrial rhythms with low RR variability to limit QTc variability caused by selecting specific RR intervals on which to average. The complete list of diagnoses used to define the *liberal* and *restrictive* subsets is available in Supplemental Table S1.

### Dependent Variable and Gold Standard

The outcome (dependent) variable was the QTc calculated using Bazett’s correction, defined as $\:QTc=QT/\sqrt{RR}$, where RR is the time between two consecutive R waves. QTc values manually-measured and overread by a PEP were considered the gold standard.

### DNN QTc Estimation

**Fig. 2:**
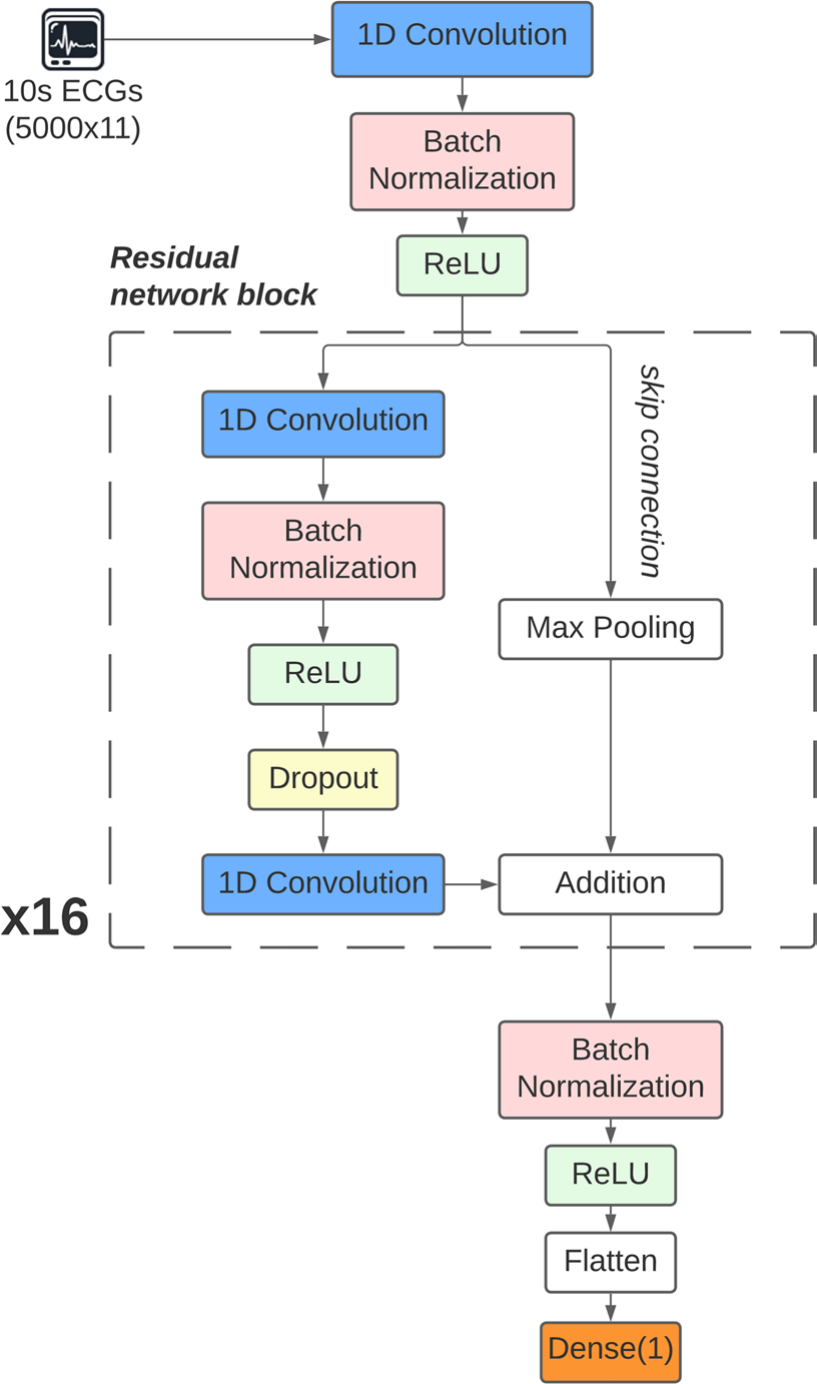
Deep-learning regression architecture for QTc estimation. The input data is the 10-sec ECG waveforms from all the 11 channels (5000 × 11 array) and the output is the scalar representing the estimated QTc interval. ReLU: rectified linear unit

We built a convolution-based, residual (ResNet) DNN for estimating QTc intervals. The DNN architecture was adapted from Hannun et al., [18] and featured sixteen cascading ResNet blocks as shown in Fig. 2. Each ResNet block comprised a 1D-convolution layer followed by batch normalization, a rectified linear unit (ReLU) activation, dropout, and a second 1D-convolution layer. Additionally, a skip-connection was used to add the output of the preceding ResNet block to the output of the second 1D convolution layer in each ResNet block. These skip connections address the vanishing gradients phenomenon during training, improving performance in deeper networks. [19] The output of the final ResNet block was flattened and fully connected to a single output node that outputs the estimated QTc interval (Fig. 2).

To optimize the DNN for clinical use, we created a second (calibrated) version of the DNN model, which we named pedQTNet, by adding a calibration transformation to the DNN’s QTc estimation. This consisted of adding a positive offset to the DNN’s predictions to concurrently achieve a positive diagnostic likelihood ratio (PLR) > 5 and a negative diagnostic likelihood ratio (NLR) < 0.2 for LQTS prediction. These were considered the minimum performance thresholds for clinical utility given the frequency of false positives and the cost of failing to diagnose a potentially fatal substrate for arrhythmia. [20] The calibration derivation involved only training data to prevent data leakage. Multiple offsets were computed for different thresholds of LQTS (i.e., 460, 470, and 500 ms). Finally, a monotonic transformation was applied to QTc predictions from the hold-out set by adding the offsets calculated from training data only if the addition of said factor moved the predicted QTc above any of the LQTS thresholds. The complete procedure is described in Algorithms S1 and S2 of the supplemental material.

In addition to the calibrated model (pedQTNet), we explored models using two reduced subsets of ECG leads. The subset of leads [I, II, V5, and V6] represents the leads most commonly used for manual measurement of the QTc in clinical practice. The subset of leads [I, II, and V1] captures the leads most commonly recorded for continuous ECG monitoring of inpatients. Additionally, we analyzed each individual lead independently.

### Commercial Algorithm for QTc Estimation Baseline

As a baseline for performance comparisons, we used GE Healthcare’s Marquette 12SL (Mar12SL) analysis program, [21] which is used by the GE devices used to collect the ECGs to automatically calculate the QTc. Briefly, it calculates a median beat waveform from all beats for each lead, aligned by their QRS complexes. Then, the QT interval is defined as the interval from the earliest Q-wave onset to the latest T-wave offset, in any lead. [21]

### Performance Evaluation and Statistical Analysis

We evaluated the performance of the pedQTNet model via nested 10-fold cross validation. In each iteration of the cross validation, 90% of the dataset was used as training set to optimize DNN parameters. The remaining 10% was used as hold-out test set for model evaluation, and the training and evaluation process was repeated 10 times. Importantly, there was no patient overlap between training and test folds. For consistency, the same fold-splitting and averaging was applied to the Mar12SL QTc estimations.

Evaluation metrics for QTc estimation included mean absolute error (MAE), mean error (ME), and standard deviation of error (SDE). Additionally, we assessed the agreement between estimated and true QTc’s via Bland-Altman plots. [22] The presence of proportional bias was identified when regression slope was significantly different from zero, [23, 24] which was tested via Wald tests. [25] For LQTS classification, evaluation metrics included sensitivity, specificity, positive (PPV) and negative (NPV) predictive values, PLR, and NLR. The 95% confidence intervals of all metrics were computed via bootstrapping with 2,000 replicates in each cross-validation fold. [26] The statistical significance of differences in QTc estimate metrics was assessed with two-sided Wilcoxon signed-rank tests for ME and MAE and two-sided Bartlett tests for SDE. For LQTS classification metrics, statistical significance was assessed via McNemar tests for sensitivity and specificity, [27] a generalized score statistic proposed by Leisenring, Alonzo and Pepe for PPV and NPV, [28] a regression model approach proposed by Gu and Pepe for PLR and NLR, [29], and bootstrapped confidence intervals of differences for F1 scores.

### Model Fairness Evaluation

We compared the MAE of predicted QTc’s across different demographic subgroups by age (newborns [0 to 1 month], infants [1 to 12 months], toddlers [1 to 5 years], school-aged children [5 to 11 years] and adolescents [11–18 years]), sex (male vs. female), and race (black or African American vs. white) in the *full* dataset.

### External Validation: Prospective Cohort

For external validation of the DNN models against the clinical gold standard (i.e., PEPs), diagnostic performance was assessed on a separate prospective cohort. This cohort comprised 200 randomly-selected ECGs from 200 patients performed in 2021 at the same institution in either an outpatient or inpatient context. After redacting all aspects of ECGs except age, sex, race, ventricular rate, and the ECG waveforms, three PEPs manually measured QTc’s using digital calipers for each of the 200 ECGs. The QTc was also estimated by pedQTNet and Mar12SL. For all algorithms, the gold standard for diagnostic performance was the average of the PEPs manual measurements. For the PEPs themselves, the gold standard was the average of the group after excluding their own measurement. [30] For the prospective cohort, confidence intervals were calculated using the normal approximation of the binomial distribution without (sensitivity, specificity, and predictive values) and with (likelihood ratios) log transformation, and bootstrapping (F1 score). [31] Given the dynamic gold standard for the PEPs, comparisons between groups were performed using independent two-sample z-tests (sensitivity, specificity, NPV, PPV, NPL, PLR) and bootstrapped confidence intervals of differences (F1 score).

## Results

**Fig. 3:**
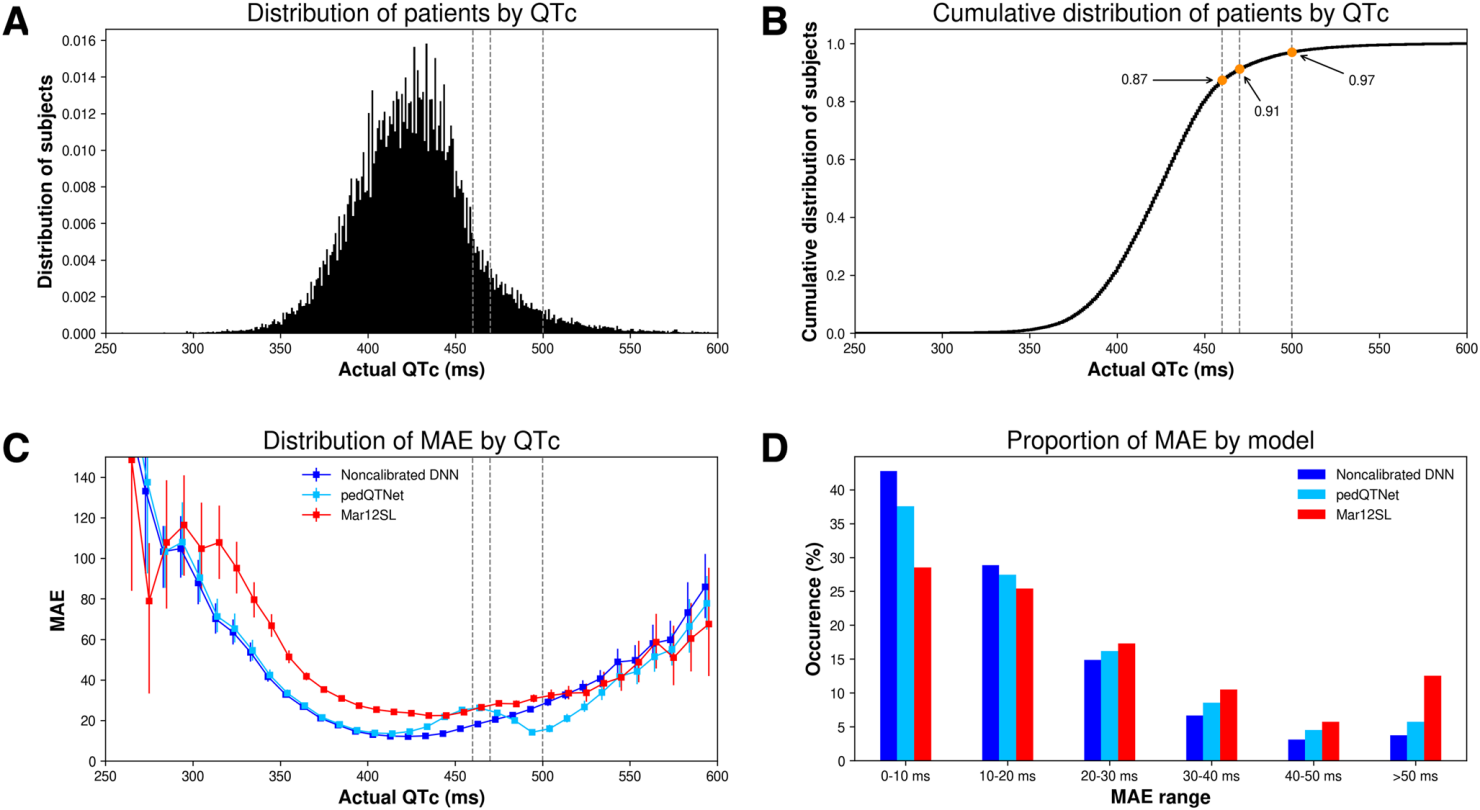
Properties of actual QTc and the mean absolute error (MAE). The distribution (**A**) and cumulative distribution (**B**) of cases according to the overread QTc are shown in the top row. The bottom row shows the MAE according to the actual QTc (**C**) and the percentage of patients for each 10-sec increasing buckets of MAE (D). In (**C**), error bars represent 95% confidence intervals. The whole (*full*) dataset, the full 10-sec ECG, and the 11 ECG leads were used for MAE estimation. For the DNNs, results represent the models trained both with (pedQTNet) and without calibration. Vertical gray lines in (**A**), (**B**), and (**C**) are the thresholds commonly adopted for classification of long QTc, i.e., 460, 470 and 500 ms. The bin resolution is 1 ms in (**A**) and (**B**), and 10 ms in (**C**). DNN: deep neural network; Mar12SL: GE’s Marquette 12SL analysis program

In total, 453,814 ECGs were obtained from 199,571 patients, of which 65,370 recordings from 37,992 patients met inclusion criteria and constituted the *full* dataset (Fig. 1). Of those ECGs, 58,324 constituted the *liberal* subset and a further 43,914 constituted the *restrictive* subset. There were no clinically-relevant differences in age or proportion of sex or race between the three datasets (Table 1). The mean QTc was 424 ms (IQR 403–445) (Fig. 3). The proportions of ECGs whose QTc was greater than 460, 470, and 500 ms were 13%, 9%, and 3%, respectively (Table 1; Fig. 3).

**Table 1:** Demographic information of the final cohort. Numerical variables are median [25th, 75th] percentile. Categorical variables are the number of subjects (% of total)

	Full	Liberal	Restrictive
Num. recordings	65,370	58,324	43,914
Age (years)	8.8 [1.4, 14.2]	9.2 [1.8, 14.3]	8.6 [1.2, 14.0]
Sex			
* Males*	33,300 (50.9)	29,375 (50.4)	22,247 (50.7)
* Females*	32,068 (49.1)	28,947 (49.6)	21,666 (49.3)
* Unknown*	2 (0.0)	2 (0.0)	1 (0.0)
Race			
* Asian*	1,762 (2.7)	1,580 (2.7)	1,231 (2.8)
* Black or African American*	16,536 (25.3)	14,889 (25.5)	10,657 (24.3)
* Other*	12,264 (18.8)	10,677 (18.3)	8,327 (19.0)
* Unknown*	628 (1.0)	533 (0.9)	408 (0.9)
* White*	34,180 (52.3)	30,645 (52.5)	23,291 (53.0)
ECG			
* Heart Rate (bpm)*	96 [77, 126]	95 [76, 124]	100 [80, 127]
* QTc Bazett (ms)*	424 [403, 445]	424 [402, 444]	425 [404, 444]
* QTc ≥ 460*	8,575 (13.1)	7,068 (12.1)	5,411 (12.3)
* QTc ≥ 470*	5,910 (9.0)	4,793 (8.2)	3,691 (8.4)
* QTc ≥ 500*	1,973 (3.0)	1,531 (2.6)	1,149 (2.6)
Diagnosis*			
* Structural Congenital Heart Disease*	17,250 (26.4)	14,437 (24.8)	11,840 (27.0)
* Myocarditis*	787 (1.2)	661 (1.1)	440 (1.0)
* Cardiomyopathy*	3,891 (6.0)	3160 (5.4)	2396 (5.5)
* Arrhythmias*	22,655 (34.7)	18,598 (31.9)	13,907 (31.7)
* Heart failure*	5,286 (8.1)	4,131 (7.1)	3,223 (7.3)
* Other*	61,981 (94.8)	55,208 (94.7)	41,693 (94.9)
* Unknown*	2,312 (3.5)	2,161 (3.7)	1,481 (3.4)

### Estimation of QTc Values

In the full dataset, the best QTc estimation was achieved by the noncalibrated DNN with an MAE of 16.2 (95% CI: 15.8-16.6) ms. The calibration of pedQTNet prolonged the noncalibrated DNN’s estimated QTc when close to the prediction thresholds for LQTS, thereby increasing the MAE to 18.8 (18.4–19.2) ms. While pedQTNet had a slightly higher MAE compared to the noncalibrated DNN, it outperformed Mar12SL, which achieved an MAE of 27.0 (26.3–27.7) ms. These differences were statistically significant, as shown in Table 2. Regarding mean errors (MEs), the noncalibrated DNN overestimated QTc’s by a negligible 0.6 ms, whereas the calibrated pedQTNet overestimated QTc’s by 5.8 ms. This overestimation, however, is clinically insignificant, especially relative to Mar12SL’s QTc overestimation of 19.3 ms. Similar results were obtained for the liberal and restrictive dataset, as shown in Table S2.

**Table 2:** QTc estimation performance for the Mar12SL, noncalibrated DNN and pedQTNet algorithms, applied to the *full* dataset using all 11 ECG leads

	Mar12SL	Noncalibrated DNN	pedQTNet*
MAE (95% CI)	27.0 (26.3 to 27.7)	16.2 (15.8 to 16.6)	18.8 (18.4 to 19.2)
ME (95% CI)	-19.3 (-20.1 to -18.5)	-0.6 (-1.2 to -0.1)	-5.8 (-6.4 to -5.2)
SDE (95% CI)	34.7 (33.3 to 36.1)	22.5 (21.8 to 23.3)	25.2 (24.4 to 25.9)

*DNN* deep neural network, *Mar12SL* GE Healthcare’s Marquette 12SL analysis program, *CI* confidence interval, *MAE *mean absolute error, *ME* mean error, *SDE* standard deviation of error. All metrics are in milliseconds. *pedQTNet is the reference model for statistical significance testing. Mar12SL and Noncalibrated DNN metrics are significantly different from pedQTNet (*p*-value < 0.05) for both datasets

The performance of DNN variants (individual leads and leads subsets) are available in Supplemental Table S3. Consistent with typical clinical practice, lead II showed the best individual MAE for the noncalibrated DNN. No combination of leads substantially improved observed MAEs. However, the best overall performance was obtained using all leads. For pedQTNet, the MAE using all leads (18.8 [18.4–19.2] ms) was equal to the MAE using the best single lead (18.8 [18.4–19.3] ms, lead I).

The MAE as a function of the gold standard QTc followed a U-shaped curve for all algorithms, with maximal performance in the range of QTc’s most seen clinically (400–450 ms; Fig. 3C). Notably, pedQTNet achieved its lowest errors and outperformed the remaining algorithms by the largest margin in the most clinically-relevant pathologic range of QTc’s (470–530 ms). The MAE of the DNN models was within 0–10 ms for more than 35% of ECGs, and within 20 ms for more than 60% of ECGs (Fig. 3D).

**Fig. 4:**
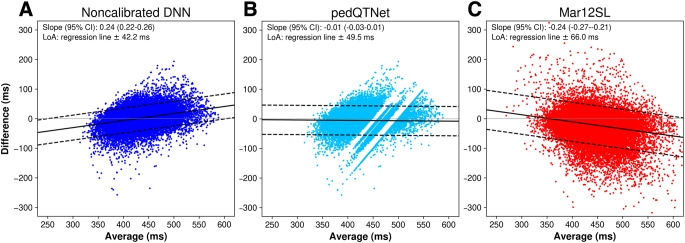
Bland-Altman plots showing the actual QTc (X-axis) and the difference between the actual QTc and the estimated QTc (Y-axis) given by the noncalibrated DNN (**A**), pedQTNet (**B**), and Mar12SL (**C**) methods. The regression line and its 95% limits of agreement (black lines) are illustrated, as well as the reference null difference (gray line). Results are shown for the *full* dataset. For the range of values shown in the plots, 13 points are not visible for Mar12SL (< 0.02%). Slopes for noncalibrated DNN and Mar12SL are statistically different from zero (Wald’s Test with t-distribution of the test statistic) [32]. LoA: limits of agreement; DNN: deep neural network; Mar12SL: GE’s Marquette 12SL analysis program

Bland-Altman plots for the noncalibrated DNN, pedQTNet, and Mar12SL algorithms as compared to the manually measured QTc using the *full* dataset are shown in Fig. 4, and plots for the *liberal* and *restrictive* datasets can be found in Fig. S1 of the Supplemental Material. The regression slopes in the Bland-Altman plots indicate that the noncalibrated DNN overestimated short QTc’s and underestimated long QTc’s, while the opposite happened with Mar12SL. In contrast, pedQTNet did not show dynamic bias as the slope of the regression line was not statistically different than zero. The empty bands observed in the pedQTNet plot (Fig. 4B) represent the calibration factor added to borderline model predictions close to LQTS thresholds.

### Model Fairness Evaluation

MAE differences of pedQTNet estimated QTc’s between sexes were not statistically significant (18.9 [18.7–19.1] vs. 18.7 [18.5–18.9], *P* = 0.16). For black and white race groups, while the MAE difference was statistically significant (18.1 [17.8–18.4] vs. 18.5 [18.4–18.7], *P* = 0.04), a difference of 0.4 ms is clinically irrelevant. MAEs were higher (*P* < 0.05) in neonates, infants, and toddlers (24.7 [24.1–25.3], 21.5 [21.1–21.8], and 20.2 [19.8–20.5] ms, respectively) than in older children (16.9 [16.7–17.2] and 18.8 [18.7–18.9] ms in 5–11 and 11–18 year olds, respectively), consistent with an increase in MAEs at higher heart rates (Figures S4-5 in the Supplemental Material).

### Classification of LQTS

At LQTS cutoffs of ≥460, ≥470, and ≥500 ms, the noncalibrated DNN yielded the highest specificity (97–99%), PPV (68–73%), PLR (17.93–71.98), and F1 score (0.49–0.64), at the expense of a lower sensitivity (39–57%) and worse NLR (0.44–0.61) for the full dataset (Table 3). The Mar12SL algorithm leaned in the opposite direction, achieving high sensitivity (75–85%) and a low NLR (0.19–0.26) at the expense of PLR (3.97–10.48). In contrast, pedQTNet achieved a good balance between NLR (0.16–0.19) and PLR (5.85–9.47). Because of its dynamic calibration, pedQTNet’s NLR improved as the QTc becomes more pathologic (0.16 for QTc > 500 ms vs. 0.19 for QTc > 460 ms), whereas the noncalibrated DNN and Mar12SL’s NLR worsened at detecting markedly prolonged QTc’s. Similar results were obtained for the liberal and restrictive dataset, as shown in Table S4.

**Table 3:** Long QTc classification performances for the Mar12SL, noncalibrated DNN, and pedQTNet algorithms, using the *full* dataset, full 10-sec ECG recordings, and all 11 leads

		Mar12SL	Noncalibrated DNN	pedQTNet *
QTc ≥ 460	**Sens**	0.85 (0.84–0.87)	0.57 (0.55–0.59)	0.84 (0.82–0.85)
	**Spec**	0.78 (0.78–0.79)	0.97 (0.96–0.97)	0.86 (0.85–0.86)
	**PPV**	0.37 (0.36–0.39)	0.73 (0.71–0.75)	0.47 (0.45–0.48)
	**NPV**	**0.97 (0.97–0.98)** ^**a**^	0.94 (0.93–0.94)	0.97 (0.97–0.97)
	**PLR**	3.97 (3.85–4.11)	17.93 (16.40–19.68)	5.85 (5.62–6.09)
	**NLR**	0.19 (0.17–0.20)^a^	0.44 (0.42–0.46)	0.19 (0.17–0.21)
	**F1**	0.52 (0.51–0.53)	0.64 (0.62–0.65)	0.60 (0.59–0.61)
QTc ≥ 470	**Sens**	0.83 (0.81–0.85)	0.53 (0.51–0.56)	0.85 (0.83–0.87)
	**Spec**	0.84 (0.84–0.85)	0.98 (0.98–0.98)	0.87 (0.87–0.88)
	**PPV**	0.35 (0.33–0.36)	0.72 (0.70–0.75)	0.40 (0.38–0.42)
	**NPV**	0.98 (0.98–0.98)	0.95 (0.95–0.96)	0.98 (0.98–0.99)
	**PLR**	5.37 (5.16–5.59)	26.83 (24.03–30.10)	6.73 (6.45–7.03)
	**NLR**	0.20 (0.18–0.22)	0.48 (0.45–0.50)	0.17 (0.15–0.19)
	**F1**	0.49 (0.47–0.51)	0.61 (0.59–0.63)	0.54 (0.53–0.56)
QTc ≥ 500	**Sens**	0.75 (0.72–0.79)	0.39 (0.35–0.43)	0.85 (0.82–0.88)
	**Spec**	0.93 (0.92–0.93)	0.99 (0.99–1.00)	0.91 (0.90–0.91)
	**PPV**	0.25 (0.23–0.26)	0.68 (0.63–0.73)	0.23 (0.21–0.24)
	**NPV**	0.99 (0.99–0.99)	0.98 (0.98–0.98)	0.99 (0.99–1.00)
	**PLR**	10.48 (9.78–11.22)	71.98 (58.29–91.32)	9.47 (8.95–10.02)
	**NLR**	0.26 (0.23–0.30)	0.61 (0.57–0.65)	0.16 (0.13–0.20)
	**F1**	0.37 (0.35–0.39)	0.49 (0.45–0.53)	0.36 (0.33–0.38)

*DNN* deep neural network, *Mar12SL* GE Healthcare’s Marquette 12SL analysis program, *WT* wavelet transform, *Sens* sensitivity, *Spec *specificity, *PPV* positive predictive value (precision), *NPV* negative predictive value, *PLR* positive likelihood ratio, *NLR* negative likelihood ratio; * pedQTNet is the reference model for statistical significance testing; ^**a**^*p*-value > 0.05 (nonsignificant) compared to pedQTNet. *P*-value for all comparisons between models excluding the pedQTNet are < 0.05

### External Validation Using a Prospective Cohort

Fourteen (7%) and twelve (6%) of ECGs had QTc’s longer than 460 and 470 ms, respectively. The performance of pedQTNet was validated in the prospective cohort (Table 4). The observed MAE of 16.0 (14.1–18.1) ms was not statistically-significantly different than that of PEPs, who achieved an MAE of 17.3 (16.0-18.5) ms (*P* = 0.14). PEPs demonstrated the highest specificities (96–98%) and resultant PLRs (20.2–40.0) but did not achieve the 0.2 NLR threshold (0.21–0.28) at either the ≥ 460 or ≥ 470 ms cutoffs. Conversely, pedQTNet and the Mar12SL algorithm achieved their screening mandates with perfect sensitivities and NLRs for both cutoffs with no significant differences between the algorithms on these metrics. However, the Mar12SL algorithm achieved this sensitivity by overestimating the true QTc, with a MAE of 30.3 (26.5–34.2) ms, which was significantly higher than that of pedQTNet and PEPs (*P* < 0.05). The corresponding p-values comparing PEPs, Mar12SL and pedQTNet are shown in Table S5.

**Table 4:** Mean error, mean absolute error, and diagnostic performance metrics for Long QT classification for the pediatric electrophysiologists (PEP), the Marquette 12SL algorithm, and pedQTNet for the 200-ECG prospective cohort

		PEPs(*n* = 600)	Mar12SL(*n* = 200)	pedQTNet(*n* = 200)
Mean Error		*n*/a	-25.2 (-29.7 to -20.8)	-12.0 (-14.5 to -9.6)^b^
Mean Absolute Error		17.3 (16.0–18.5)	30.3 (26.5–34.2)^a^	16.0 (14.1–18.1)^b^
QTc ≥ 460	**Sens**	0.72 (0.56–0.85)	1.00 (0.75–1.00)^a^	1.00 (0.75–1.00)^a^
	**Spec**	0.96 (0.95–0.98)	0.80 (0.73–0.85)^a^	0.86 (0.80–0.91)^a^
	**PPV**	0.61 (0.46–0.74)	0.25 (0.14–0.40)^a^	0.33 (0.19–0.50)^a^
	**NPV**	0.98 (0.96–0.99)	1.00 (0.98–1.00)^a^	1.00 (0.98–1.00)^a^
	**PLR**	20.1 (12.6–32.1)	4.9 (3.7–6.5)^a^	7.2 (5.0–10.3)^a, b^
	**NLR**	0.29 (0.18–0.47)	0.0 (0.0–0.68)^c^	0.00 (0.00–0.63)^a^
	**F1**	0.66 (0.53–0.76)	0.41 (0.24–0.55)^a^	0.50 (0.31–0.65)^b^
QTc ≥ 470	**Sens**	0.71 (0.53–0.85)	1.00 (0.69–1.00)^a^	1.00 (0.69–1.00)^a^
	**Spec**	0.98 (0.97–0.99)	0.88 (0.82–0.92)^a^	0.88 (0.83–0.93)^a^
	**PPV**	0.71 (0.53–0.85)	0.30 (0.16–0.49)^a^	0.31 (0.16–0.50)^a^
	**NPV**	0.98 (0.97–0.99)	1.00 (0.98–1.00)^a^	1.00 (0.98–1.00)^a^
	**PLR**	40.0 (20.8–76.7)	8.3 (5.6–12.1)^a^	8.6 (5.8–12.8)^a^
	**NLR**	0.30 (0.18–0.50)	0.0 (0.0–0.78)^c^	0.0 (0.0–0.77)^c^
	**F1**	0.71 (0.57–0.82)	0.47 (0.26–0.64)^a^	0.48 (0.26–0.64)^a^

*PEP* pediatric electrophysiologists, *DNN* deep neural network, *Sens* sensitivity, *Spec* specificity, *PPV* and *NPV* positive and negative predictive values, *PLR* and *NLR* positive and negative likelihood ratios. ^a^*p*-value < 0.05 compared to PEPs; ^b^*p*-value < 0.05 compared to Marquette 12SL algorithm. Pairwise *p*-values are in supplemental Table S3. The mean error of the PEPs is zero by definition (n/a). ^c^A smoothing constant of 0.5 was added to the contingency matrix in the event of undefined metric

## Discussion

In this study, we developed a fully automated deep-learning approach and demonstrated the clinical utility of DNN models to estimate the QTc in pediatric ECGs. In pursuing clinical usability, we developed a calibrated DNN (pedQTNet) for LQTS classification. The noncalibrated DNN achieved an MAE 11 ms lower than a widely-used commercial algorithm. However, this precision came at the cost of detection performance of LQTS. Thus, pedQTNet was developed with the dual mandate of achieving a NLR of 0.2 to reliably rule out LQTS at three different cutoffs (460, 470, and 500 ms) and a PLR of 5 to prevent excessive false positives. In the initial validation on the *full* dataset at a cutoff of 470 ms, both objectives were met (NLR 0.17, PLR 6.7) while maintaining a MAE of 18.8 ms. This performance improved further in the prospective cohort and in so doing matched or outperformed experts (PEPs) as a screening test. While PEPs achieved the highest PLRs in the prospective cohort, their ability to detect LQTS lagged the calibrated DNN with lower sensitivity and insufficient NLR.

Further, the calibrated model predicted the mean QTc of three PEPs better than any individual PEP could predict the consensus of their peers. This highlights the ability of some machine-learning approaches to outperform their own labels when there is random error in the labels. [33] However, given the limited number of prospective ECG recordings and 3 PEPs, further evaluation should be considered.

We prioritized the NLR as the most clinically-relevant for an algorithm meant as a screening tool. The primary clinical use cases for the application of pedQTNet would be in emergency rooms, non-cardiac inpatient units, and lower-resource institutions that lack PEPs. In these settings, the ability to reliably produce low post-test probabilities of long QT is of paramount importance, and the NLR informs this objective. Notably, Mar12SL’s algorithm is also designed to be conservative, and has similar NLRs as our pedQTNet. These high detection rates are, however, achieved only with excessive overestimation of true QTc’s and high false-positive rates, which substantially limit its clinical use. Mar12SL’s algorithm is further limited by a NLR which worsens as the underlying QTc prolongs beyond 470 and 500 ms, an undesirable quality given that the risk of arrhythmia increases dramatically at markedly long QTc’s.

The Mar12SL algorithm’s performance in the validation and prospective cohorts significantly underperformed relative to previously-published results. [34] This may reflect challenges in measuring QTc’s in pediatric populations, the inclusion of abnormal ECGs in this study, differences in preprocessing, or the method of QT measurement. Specifically, PEPs in practice measure QTc’s on specific leads using the tangent method rather than median waveforms of all leads as the Mar12SL algorithm does. This is not to say that one method is superior, but reference ranges would need to account for what are likely true and material differences between these methods.

Gold standard labels were limited to QTc’s measured manually by PEPs. While using computer-measured QTc’s attractively increases the size of the training and development sets, the resulting algorithms learn to predict not the expert human measurement but the computerized QTc measurement with all the attendant limitations discussed herein. Limiting the readers to PEPs who worked at quaternary care children’s hospitals ensured their expertise both in inherited arrhythmias and the specific challenges of pediatric ECG interpretation. QTc measurement—even by content experts—still results in some interrater variability, which was mitigated in the prospective cohort by using a consensus measurement. pedQTNet demonstrated performance fairness across sex and race. However, there were performance differences across age groups, which should be addressed in follow-up studies. Performance differences across demographic categories were more pronounced for the Mar12SL algorithm (Figures S2, S3, and S4).

Finally, the proposed DNN models are robust for situations where limited ECG leads are available, such as inpatient telemetry or ambulatory ECG monitoring. For example, lead II alone achieved a MAE within 1 ms compared to all leads combined. The DNN models are also robust across a wide range of electrocardiographic abnormalities, including non-sinus rhythms, with trivial improvements in MAE performance even using the most restrictive cohort.

This study builds upon previous work in the DNN-estimated-LQTS space by optimizing clinical usability and by specifically serving the pediatric population. In a recent study, Giudicessi et al. utilized a 2-dimensional DNN to predict the QTc interval from the ECG in an adult population (mean 61 years of age) which they then applied to 2-lead ECGs from a mobile, retail device [16]. When comparing performance in the prospective cohort, the pedQTNet’s NLR exceeds that of the DNN (0.28–0.33), reflecting our model’s innovative optimization for clinical screening. Additional work has demonstrated the ability of deep neural networks to detect genotype positive LQTS even in individuals in whom the QTc is not prolonged. [17, 35] Given the risk of TdP in markedly prolonged QTc’s even in acquired LQTS, we deliberately trained this model to detect LQTS irrespective of etiology.

### Limitations

This study has limitations. First, we validated the pedQTNet model in a single-center cohort. External validation is needed to assess the generalizability to other geographical regions. Second, while a strength of the current model is that only QTc’s manually measured by a cardiologist were used for training data, not all QTc’s measured during clinical care are measured as carefully as those performed specifically for long QTc. This may help explain the superior MAE achieved on the prospective cohort. While the training and validation sets are large, the human resources required for dedicated manual annotation of QTc measurements limited the size of the prospective cohort to 200 ECGs, in turn limiting the power to detect true but small diagnostic performance differences. Additional evaluation of the model in larger external datasets remain the focus of future work. Third, our model architecture learned from ‘raw’ ECG voltages provided by Mar12SL, and additional validation on other vendors’ devices may be necessary due to potentially variable voltage-preprocessing platforms. Many DNN models proposed in the literature for ECG analysis require pre-processing steps after the individual lead voltages are attained, e.g., using third-party median waveforms or RR intervals. [12, 14, 16, 36–39] In contrast, our DNNs use the entire 10-sec ECG as input, requiring neither beat segmentation nor median waveforms. We believe this strategy may improve generalizability and accuracy.

### Future Work

An inherent limitation of DNNs is their limited explainability. While recent advances in explainable artificial intelligence (e.g., ECGradCAM [40]) can indicate the ECG inputs most important to the DNN, simply indicating that the QT interval is important to estimating the QTc is of limited value. Higher value future studies should instead focus on externally validating the calibrated DNN’s prediction of the QTc itself and, preferably, the risk of ventricular arrhythmias in a multi-center cohort.

The DNN architecture used in this study was an extension of the work of Hannun et al., whose DNN classified different rhythms from the ECG. [18] Nevertheless, similar residual DNN architectures have been used in different problems involving ECG waveforms, suggesting the high potential of this architecture in different scenarios such as recognition of ECG abnormalities, knowledge discovery, and arrhythmia detection. [11, 40, 41] We believe this model could be extended to estimation of other ECG features including PR segments, ST elevation, or T-wave morphologies to automate monitoring of conduction delay, ischemia, or ventricular strain on both resting ECGs and—with transfer learning of model weights—to continuous telemetry. Such models could add great value to algorithms that process routine continuous bedside monitor data, serving as the inputs to models which predict clinical deterioration in ICUs (e.g., cardiac arrest) or drug-induced adverse events (e.g., QTc prolongation from chemotherapy agents). [42, 43]

## Conclusion

We developed a deep-learning algorithm optimized for a pediatric clinical application that estimated the QTc with greater accuracy than a current commercial algorithm. Our model demonstrated the potential to perform at least at the level of expert human readers (PEPs). By achieving higher sensitivity to detect either congenital or acquired LQTS compared to human readers and higher positive likelihood ratios compared to current commercial algorithms, our proposed calibrated DNN may be a viable option for large-scale computerized QTc monitoring and risk stratification.

## Supplementary Information

Below is the link to the electronic supplementary material.

Supplementary Material 1 (PDF 2.39 MB)

## Data Availability

The datasets analyzed during the current study are not publicly available due to patient privacy concerns. Sharing of study data is subject to the establishment of data-sharing agreements between institutions, approved by the Children’s Hospital of Philadelphia.
