# Supplementary material for: pedQTNet: A Deep Learning Approach to Estimate Corrected QT Intervals from Multi-Lead Conventional ECG Waveforms in Pediatric Patients
Source: J Med Syst. 2026 Jun 3;50(1):90. doi: 10.1007/s10916-026-02386-1 (PMC13233968; doi:10.1007/s10916-026-02386-1)
Supplement: Supplementary file 1 — Supplementary Material 1 (PDF 2.39 MB) [file 10916_2026_2386_MOESM1_ESM.pdf]

Supplemental Material

TABLES

Table S1. Diagnosis inclusion and exclusion criteria for the *Liberal* and *Restrictive* subsets.

Diagnosis Label	Liberal	Restrictive
Ventricular-paced rhythm	Exclude	Exclude
Non-specific T wave abnormality	Include	Exclude
Accelerated junctional rhythm	Include	Include
Artifact/Baseline wander/Poor quality	Exclude	Exclude
Baseline artifact/wander	Exclude	Exclude
Poor data quality	Exclude	Exclude
T wave flattening	Include	Exclude
Undetermined rhythm	Exclude	Exclude
Atrial fibrillation	Include	Exclude
Atrial flutter	Include	Exclude
Atrial-paced rhythm	Include	Include
Atrial rhythm	Include	Include
Atrial tachycardia	Include	Exclude
Incomplete Study	Exclude	Exclude
Lead Reversal	Exclude	Exclude
Atrial bradycardia	Include	Include
1st Degree AV block	Include	Include
2nd Degree AV block	Include	Exclude
3rd Degree AV block	Include	Include
Ectopic Atrial Rhythm	Include	Include
Ectopic Atrial Tachycardia	Include	Exclude
Premature Atrial Contractions	Include	Exclude
Premature Ventricular Contractions	Exclude	Exclude
Low right atrial rhythm	Include	Exclude
Junctional bradycardia	Include	Include
Junctional ectopic tachycardia	Include	Exclude
Ectopic atrial rhythm	Include	Include
Sinus tachycardia	Include	Exclude
Sinus arrhythmia	Include	Exclude
Sinus bradycardia	Include	Include
Supraventricular tachycardia	Include	Exclude
Non-specific ST abnormality	Include	Exclude
Normal sinus rhythm	Include	Include
Short PR interval	Include	Include
Atrial-paced ventricular-paced rhythm	Exclude	Exclude
Atrial-sensed ventricular-paced rhythm	Exclude	Exclude
ST abnormality	Include	Exclude
Wandering atrial pacemaker	Include	Exclude
Wide QRS rhythm	Exclude	Exclude
Wide QRS tachycardia	Exclude	Exclude

Table S2. QTc estimation performance for the Mar12SL, noncalibrated DNN and pedQTNet algorithms, applied to the *liberal* and *restrictive* datasets using all 11 ECG leads.

	**Mar12SL**	**Noncalibrated DNN**	**pedQTNet***
Liberal			
**MAE (95% CI)**	26.0 (25.3 to 26.8)	15.6 (15.3 to 16.0)	18.2 (17.8 to 18.7)
**ME (95% CI)**	-18.9 (-19.8 to -18.1)	-0.8 (-1.3 to -0.2)	-5.8 (-6.4 to -5.2)
**SDE (95% CI)**	32.9 (31.6 to 34.4)	21.6 (20.8 to 22.3)	24.2 (23.5 to 25.0)
Restrictive			
**MAE (95% CI)**	26.2 (25.4 to 27.0)	15.4 (14.9 to 15.8)	18.1 (17.7 to 18.7)
**ME (95% CI)**	-20.1 (-21.0 to -19.1)	-1.3 (-2.0 to -0.7)	-6.6 (-7.3 to -5.9)
**SDE (95% CI)**	32.1 (30.5 to 33.7)	21.1 (20.3 to 22.0)	23.9 (23.0 to 24.8)

DNN: deep neural network; Mar12SL: GE Healthcare's Marquette 12SL analysis program; CI: confidence interval. MAE: mean absolute error; ME: mean error; SDE: standard deviation of error. All metrics are in milliseconds. *pedQTNet is the reference model for statistical significance testing. Mar12SL and Noncalibrated DNN metrics are significantly different from pedQTNet (p-value < 0.05) for both datasets.

Table S3. Mean absolute errors (95 % confidence intervals) for QTc estimation using the DNN and WT-based methods for the *full* dataset and for each ECG lead and combination of leads.

Lead	Noncalibrated DNN	pedQTNet	WT-based
**I**	17.8 (17.4 - 18.2)	**18.8 (18.4 - 19.3)**	56.5 (55.2 - 57.8)
**II**	16.7 (16.3 - 17.1)	19.4 (18.9 - 19.8)	49.9 (48.6 - 51.2)
**V1**	18.3 (17.9 - 18.7)	19.7 (19.3 - 20.2)	87.2 (85.4 - 88.9)
**V2**	18.2 (17.8 - 18.6)	19.5 (19.1 - 20.0)	66.5 (65.1 - 68.0)
**V3**	17.7 (17.3 - 18.1)	20.4 (20.0 - 20.9)	64.3 (63.0 - 65.5)
**V3R**	18.9 (18.5 - 19.4)	18.9 (18.5 - 19.4)	95.6 (93.8 - 97.4)
**V4**	17.6 (17.2 - 18.0)	20.3 (19.8 - 20.7)	55.7 (54.5 - 57.0)
**V4R**	19.1 (18.7 - 19.6)	19.1 (18.7 - 19.6)	111.4 (109.5 - 113.3)
**V5**	16.8 (16.4 - 17.2)	19.4 (19.0 - 19.9)	45.9 (44.7 - 47.2)
**V6**	17.1 (16.7 - 17.6)	20.0 (19.5 - 20.4)	**41.0 (39.8 - 42.3)**
**V7**	17.3 (16.9 - 17.7)	19.1 (18.6 - 19.5)	39.6 (38.3 - 40.9)
**I, II, V5, V6**	16.4 (16.0 - 16.7)	19.2 (18.8 - 19.6)	44.9 (43.6 - 46.1)
**I, II, V1**	16.7 (16.3 - 17.0)	19.4 (18.9 - 19.8)	46.2 (44.9 – 47.5)
**All leads**	**16.2 (15.8 - 16.6)**	**18.8 (18.4 - 19.2)**	41.1 (40.0 - 42.2)

DNN: deep neural network; WT: wavelet transform. All metrics are in milliseconds. Mar12SL does not provide QTc estimates for each lead. DNN results are shown for both noncalibrated and calibrated (pedQTNet) models.

Table S4. Long QTc classification performances for the Mar12SL, noncalibrated DNN, and pedQTNet algorithms, using the *liberal* and *restrictive* datasets, the full 10-sec ECG recordings, and all 11 leads.

		Mar12SL	Noncalibrated DNN	pedQTNet *
Liberal				
**QTc >= 460**	**Sens**	0.86 (0.84 - 0.87)	0.57 (0.55 - 0.59)	0.84 (0.83 - 0.86)
	**Spec**	0.79 (0.79 - 0.80)	0.97 (0.97 - 0.97)	0.86 (0.85 - 0.87)
	**PPV**	0.36 (0.35 - 0.38)	0.73 (0.71 - 0.75)	0.45 (0.44 - 0.47)
	**NPV**	**0.98 (0.97 - 0.98)**[a]	0.94 (0.94 - 0.95)	0.98 (0.97 - 0.98)
	**PLR**	4.14 (4.00 - 4.30)	19.74 (17.89 - 21.88)	6.09 (5.83 - 6.36)
	**NLR**	**0.18 (0.16 - 0.20)**[a]	0.44 (0.42 - 0.46)	0.18 (0.16 - 0.20)
	**F1**	0.51 (0.50 - 0.52)	0.64 (0.62 - 0.66)	0.59 (0.58 - 0.61)
**QTc >= 470**	**Sens**	0.84 (0.82 - 0.85)	0.54 (0.51 - 0.56)	0.86 (0.84 - 0.88)
	**Spec**	0.85 (0.85 - 0.86)	0.98 (0.98 - 0.98)	0.88 (0.87 - 0.88)
	**PPV**	0.34 (0.32 - 0.35)	0.73 (0.70 - 0.75)	0.39 (0.37 - 0.40)
	**NPV**	0.98 (0.98 - 0.99)	0.96 (0.96 - 0.96)	0.99 (0.98 - 0.99)
	**PLR**	5.73 (5.49 - 5.99)	30.64 (27.08 - 34.98)	7.12 (6.80 - 7.46)
	**NLR**	0.19 (0.17 - 0.22)	0.47 (0.45 - 0.50)	0.16 (0.14 - 0.18)
	**F1**	0.48 (0.46 - 0.50)	0.61 (0.59 - 0.64)	0.53 (0.52 - 0.55)
**QTc >= 500**	**Sens**	0.75 (0.71 - 0.79)	0.40 (0.35 - 0.44)	0.85 (0.82 - 0.89)
	**Spec**	0.93 (0.93 - 0.94)	0.99 (0.99 - 1.00)	0.91 (0.91 - 0.92)
	**PPV**	0.24 (0.21 - 0.26)	0.67 (0.62 - 0.73)	0.21 (0.20 - 0.23)
	**NPV**	0.99 (0.99 - 0.99)	0.98 (0.98 - 0.99)	1.00 (0.99 - 1.00)
	**PLR**	11.47 (10.62 - 12.40)	80.72 (63.61 - 104.96)	10.18 (9.56 - 10.84)
	**NLR**	0.27 (0.23 - 0.31)	0.61 (0.56 - 0.65)	0.16 (0.12 - 0.19)
	**F1**	0.36 (0.33 - 0.39)	0.49 (0.45 - 0.54)	0.34 (0.32 - 0.37)
Restrictive				
**QTc >= 460**	**Sens**	0.87 (0.86 - 0.89)	0.59 (0.57 - 0.62)	0.86 (0.84 - 0.88)
	**Spec**	0.78 (0.77 - 0.79)	0.97 (0.97 - 0.97)	0.85 (0.84 - 0.86)
	**PPV**	0.36 (0.34 - 0.37)	0.73 (0.71 - 0.75)	0.45 (0.43 - 0.47)
	**NPV**	**0.98 (0.97 - 0.98)**[a]	0.94 (0.94 - 0.95)	0.98 (0.97 - 0.98)
	**PLR**	3.99 (3.84 - 4.15)	19.77 (17.72 - 22.20)	5.81 (5.54 - 6.10)
	**NLR**	**0.16 (0.14 - 0.18)**[a]	0.42 (0.40 - 0.45)	0.16 (0.14 - 0.18)
	**F1**	0.51 (0.49 - 0.52)	0.65 (0.63 - 0.67)	0.59 (0.57 - 0.61)
**QTc >= 470**	**Sens**	0.85 (0.83 - 0.87)	0.55 (0.53 - 0.58)	0.88 (0.86 - 0.89)
	**Spec**	0.85 (0.84 - 0.85)	0.98 (0.98 - 0.98)	0.87 (0.87 - 0.88)
	**PPV**	0.34 (0.32 - 0.36)	0.73 (0.70 - 0.76)	0.39 (0.37 - 0.40)
	**NPV**	0.98 (0.98 - 0.99)	0.96 (0.96 - 0.96)	0.99 (0.98 - 0.99)
	**PLR**	5.63 (5.36 - 5.91)	30.15 (26.25 - 35.06)	6.87 (6.53 - 7.25)
	**NLR**	0.17 (0.15 - 0.20)	0.45 (0.42 - 0.48)	0.14 (0.12 - 0.17)
	**F1**	0.49 (0.47 - 0.50)	0.63 (0.60 - 0.65)	0.53 (0.51 - 0.55)
**QTc >= 500**	**Sens**	0.76 (0.71 - 0.80)	0.39 (0.34 - 0.45)	0.87 (0.83 - 0.90)
	**Spec**	0.93 (0.93 - 0.94)	0.99 (0.99 - 1.00)	0.91 (0.90 - 0.91)
	**PPV**	0.24 (0.21 - 0.26)	0.66 (0.59 - 0.72)	0.21 (0.19 - 0.23)
	**NPV**	0.99 (0.99 - 0.99)	0.98 (0.98 - 0.99)	1.00 (1.00 - 1.00)
	**PLR**	11.57 (10.58 - 12.64)	75.50 (57.99 - 102.06)	9.80 (9.14 - 10.52)
	**NLR**	0.26 (0.21 - 0.31)	0.61 (0.56 - 0.66)	0.14 (0.11 - 0.18)
	**F1**	0.36 (0.33 - 0.39)	0.49 (0.44 - 0.54)	0.33 (0.31 - 0.36)

DNN: deep neural network; Mar12SL: GE Healthcare's Marquette 12SL analysis program; WT: wavelet transform; Sens: sensitivity; Spec: specificity; PPV: positive predictive value (precision); NPV: negative predictive value; PLR: positive likelihood ratio; NLR: negative likelihood ratio; *

pedQTNet is the reference model for statistical significance testing; [a]p-value > 0.05 (nonsignificant) compared to pedQTNet. P-value for all comparisons between models excluding the pedQTNet are < 0.05.

Table S5. Statistical analysis of mean (ME) and mean absolute (MAE) errors for QTc estimation and diagnostic performance metrics for Long QT classification in the prospective cohort (200 ECGs)

		pedQTNet vs PEPs	pedQTNet vs Mar12SL	PEPs vs Mar12SL
ME		n/a	<0.001	n/a
MAE		0.14	<0.001	<0.001
**QTc >= 460**	**Sens**	<0.001	1.00	<0.001
	**Spec**	<0.001	0.10	<0.001
	**PPV**	<0.001	0.42	<0.001
	**NPV**	<0.001	1.0	0.005
	**PLR**	<0.001	0.008	<0.001
	**NLR**	<0.001	0.710	0.17
	**F1**	0.104	0.035	0.012
**QTc >= 470**	**Sens**	0.003	1.00	0.003
	**Spec**	<0.001	0.87	<0.001
	**PPV**	<0.001	0.93	<0.001
	**NPV**	0.010	1.00	0.034
	**PLR**	<0.001	0.22	<0.001
	**NLR**	0.200	0.82	0.21
	**F1**	0.028	0.917	0.025

Mar12SL: GE Healthcare's Marquette 12SL analysis program; Sens: sensitivity; PEPs: pediatric electrophysiologists; Spec: specificity; PPV: positive predictive value (precision); NPV: negative predictive value; PLR: positive likelihood ratio; NLR: negative likelihood ratio.

FIGURES

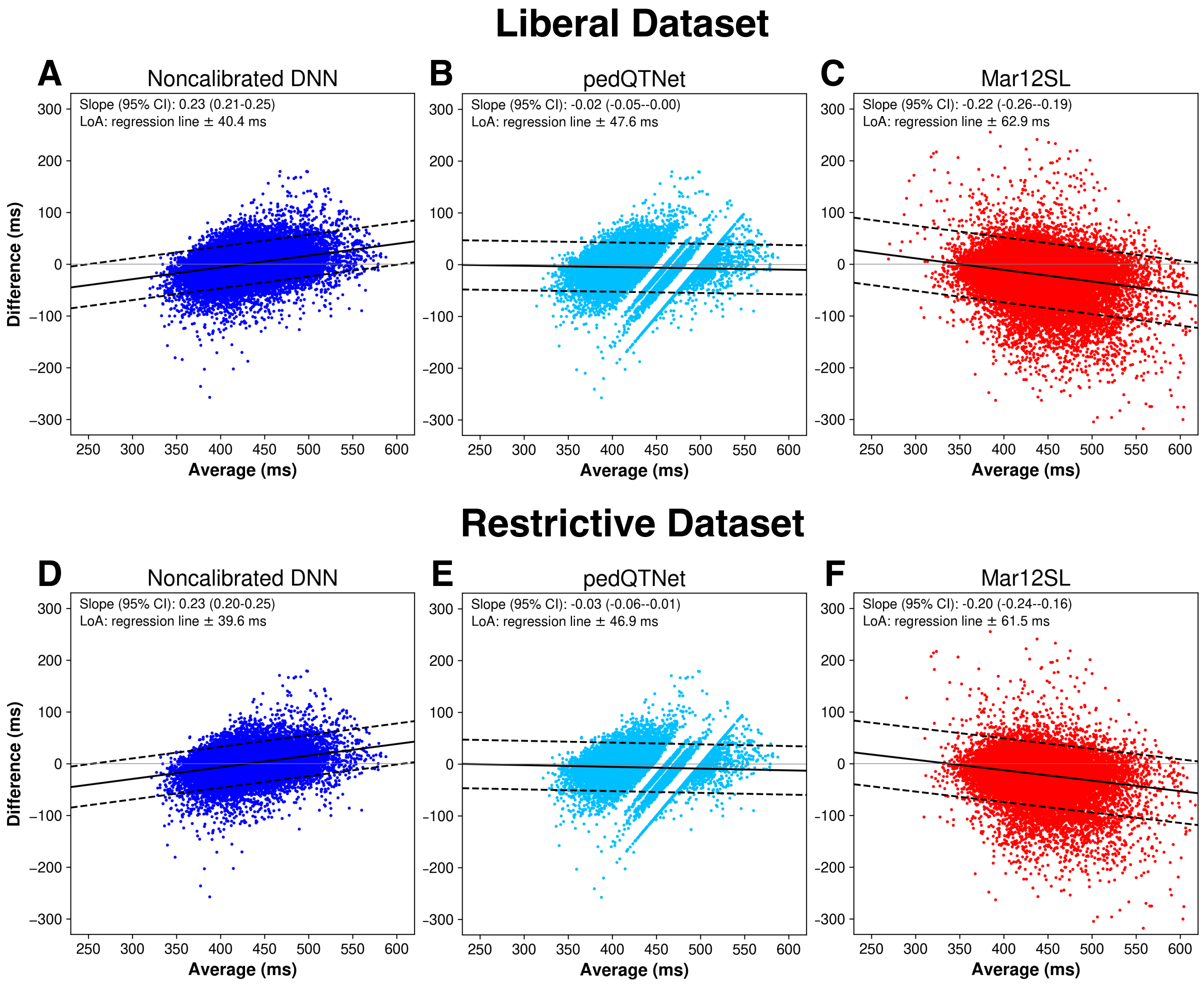

Figure S1. Bland-Altman plots showing the actual QTc (X-axis) and the difference between the actual QTc and the estimated QTc (Y-axis) given by the noncalibrated DNN (A and D), pedQTNet (B and E), and Mar12SL (C and F) methods. The regression line and the 95% limits of agreement (black lines) are illustrated. Top row represents the *liberal* dataset and bottom row the *restrictive* dataset. For the range of values shown in the plots, 9 and 7 points are not visible for the Mar12SL method for the liberal and restrictive datasets, respectively (<0.02%). All slopes are statistically different from zero (Wald's Test with t-distribution of the test statistic). DNN: deep neural network; Mar12SL: GE Healthcare's Marquette 12SL analysis program.

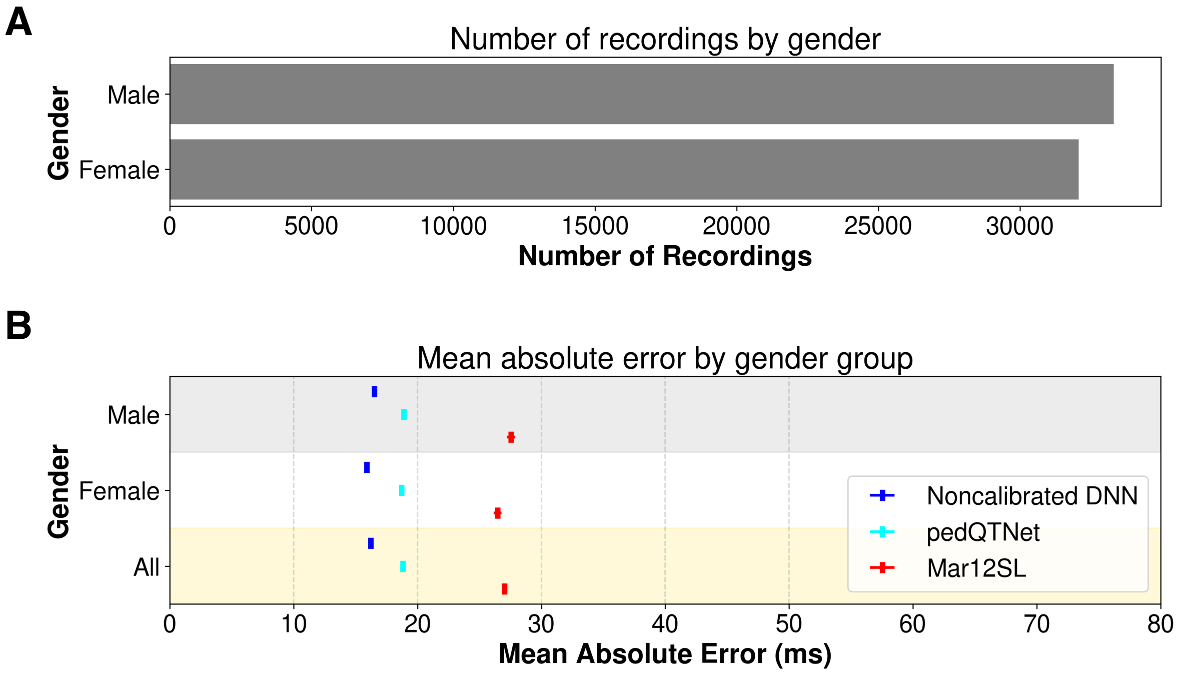

Figure S2. Number of recordings and mean absolute error (MAE) by different gender. Results for the full dataset (All) are shown for reference. In plot (B), the MAE and 95% confidence intervals are shown. Vertical reference (gray) lines are shown for MAEs of 10, 20, 30, 40, and 50 ms for comparison. The pedQTNet MAE obtained for the Male groups is not statistically different from pedQTNet MAE obtained for the Female group ($p \geq 0.05$, Mann-Whitney U rank test). DNN: deep neural network; Mar12SL: GE Healthcare's Marquette 12SL analysis program.

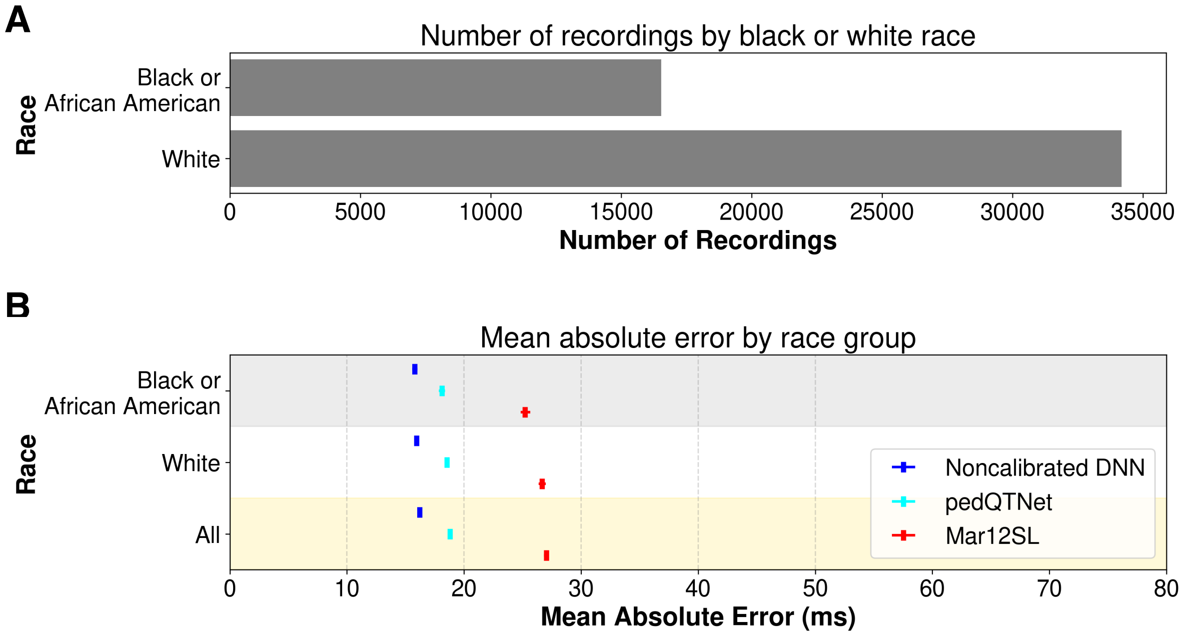

Figure S3. Number of recordings and mean absolute error (MAE) by the two majority race groups. Results for the full dataset (All) are shown for reference. In plot (B), the MAE and 95% confidence intervals are shown. Vertical reference (gray) lines are shown for MAEs of 10, 20, 30, 40, and 50 ms for comparison. The difference between White and Black/African American pedQTNet MAE was significant ($p=0.04$, Mann-Whitney U rank test), although not clinically relevant (<1 ms). DNN: deep neural network; Mar12SL: GE Healthcare's Marquette 12SL analysis program.

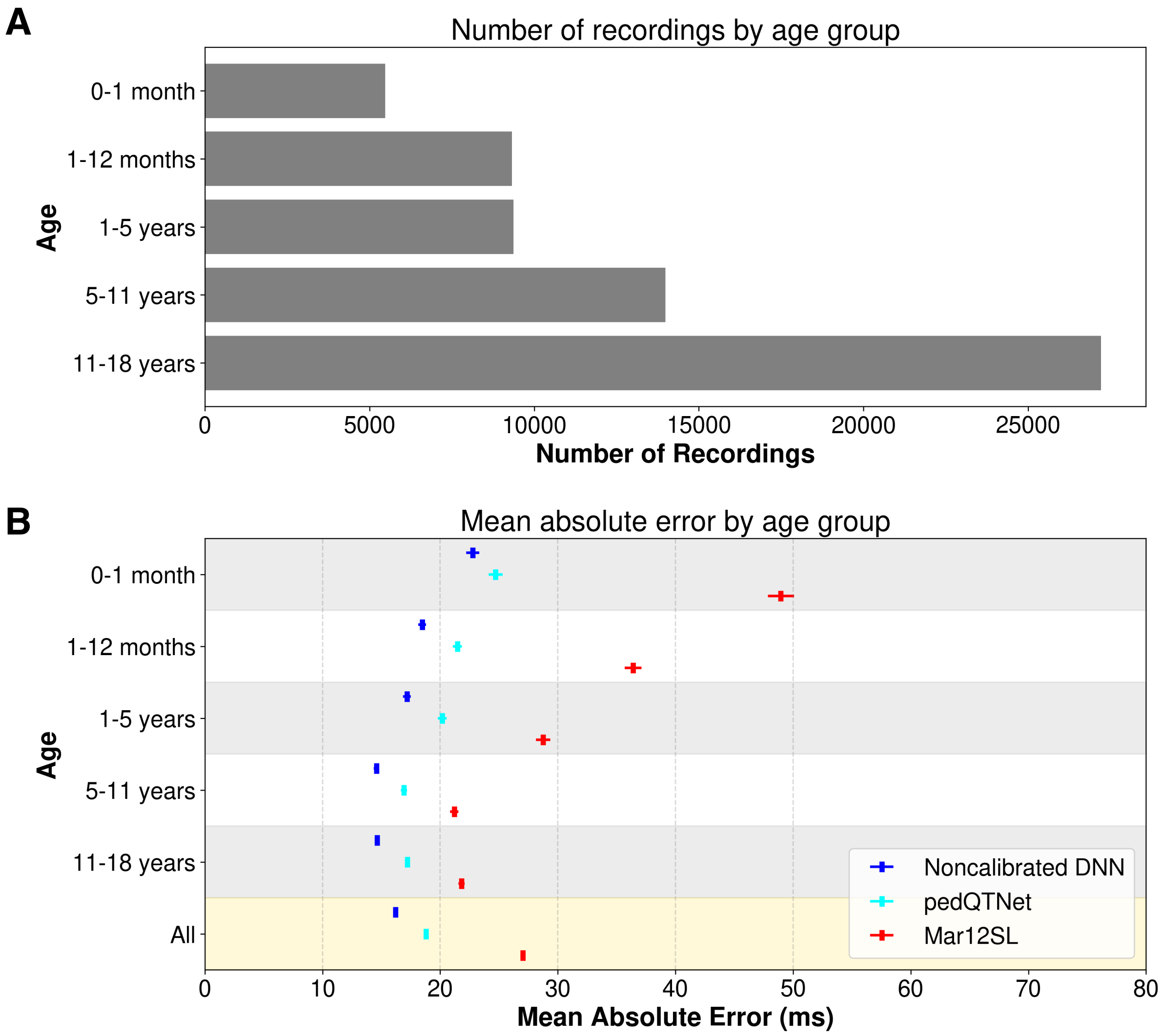

Figure S4. Number of recordings and mean absolute error (MAE) by different age groups. Results for the full dataset (All) are shown for reference. In plot (B), the MAE and 95% confidence intervals are shown. Vertical reference (gray) lines are shown for MAEs of 10, 20, 30, 40, and 50 ms for comparison. For all age ranges, lower bound was included and upper bound not included. Pairwise comparisons between all age groups for pedQTNet MAE were statistically significant ($p<0.05$), except between 5-11 years and 11-18 years groups ($p=1.0$, Mann-Whitney U rank test followed by Bonferroni correction). DNN: deep neural network; Mar12SL: GE Healthcare’s Marquette 12SL analysis program.

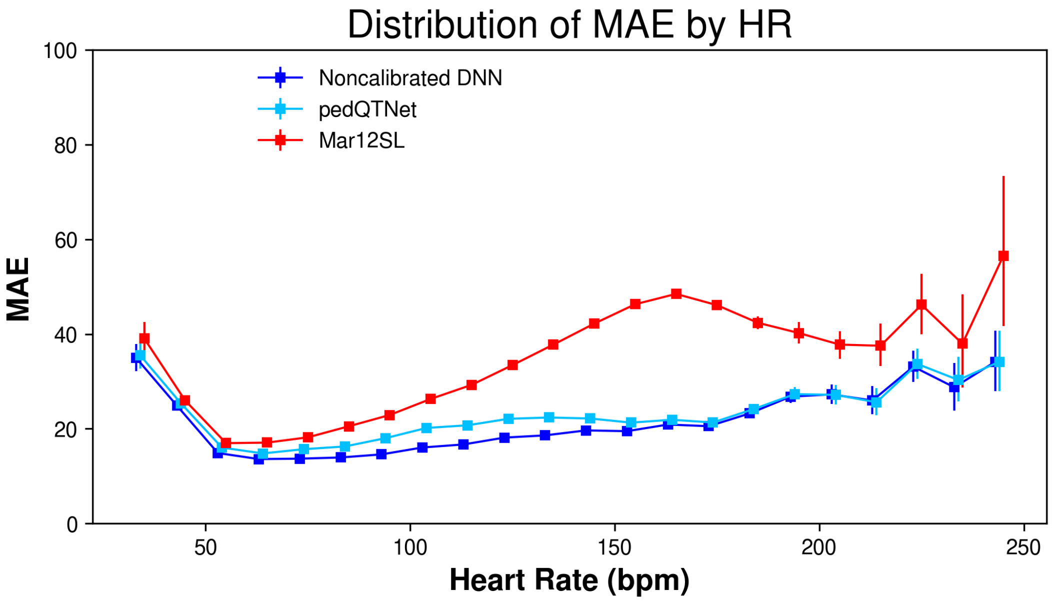

Figure S5. Distribution of mean absolute error (MAE) for different heart rates (HR). Error bars represent 95% confidence intervals. Calculations were performed over buckets of size 10 bpm in the range from 30 to 250 bpm. DNN: deep neural network; Mar12SL: GE Healthcare's Marquette 12SL analysis program.

ALGORITHMS

Algorithm S1

Bias computation algorithm

$bias = compute\_bias(QTc_{DNN}, LQTS_{threshold}, labels)$

Inputs:

$\boldsymbol{QTc_{DNN}}$: DNN regression predictions.

$\boldsymbol{LQTS_{threshold}}$: QTc threshold for dichotomizing probabilities into long QT syndrome predictions.

$\boldsymbol{labels}$: True values of QTc intervals corresponding to each DNN prediction.

Outputs:

bias: constant that, when added to DNN probabilities, aids in achieving PLR >= 5 and NLR <= 0.2.

$bias_{min} = 0$
$bias_{max} = 0$

FOR $bias_i$ in sequence(1, 50):

$predictions = \{1 \; if \; (QTc + bias_i) \geq LQTS_{threshold} \; else \; 0 \mid QTc \in QTc_{DNN}\}$

$$PLR = \frac{sensitivity(predictions, \; labels)}{(1 - specificity(predictions, \; labels))}$$

$$NLR = \frac{(1 - sensitivity(predictions, \; labels))}{specificity(predictions, \; labels)}$$

IF $PLR \geq 5 \; and \; NLR \leq 0.2$:

$bias_{max} = bias_i$

IF $bias_{min} == 0$:

$bias_{min} = bias_i$

END IF

END IF

END FOR

$$bias = \frac{(bias_{max} + bias_{min})}{2}$$

RETURN $bias$

Algorithm S2

Probability calibration algorithm:

$$QTC_{calibrated} = calibrate\_probabilities(QTc_{DNN_{train}}, QTc_{DNN_{test}}, labels_{train})$$

Inputs:

- $\boldsymbol{QTc_{DNN_{train}}}$: DNN regression predictions from training set. These will be used to derive the calibration biases.
- $\boldsymbol{QTc_{DNN_{test}}}$: DNN regression predictions from test set. These are the calibration targets.
- $\boldsymbol{labels_{train}}$: True values of QTc intervals corresponding to each sample in $\boldsymbol{QTc_{DNN_{train}}}$

Outputs:

- $\boldsymbol{QTC_{calibrated}}$: Calibrated DNN predictions

$thresholds = \{460, 470, 500\}$

$biases = \{compute\_bias(QTc_{DNN_{train}}, LQTS_{threshold}, labels_{train}) \mid LQTS_{threshold} \in thresholds\}$

$QTc_{calibrated} = []$

```
FOR i in sequence(1, length(QTc_DNN_test)):
    IF (QTc_DNN_test[i] + max(biases) < 460) or (QTc_DNN_test[i] ≥ 500):
        QTc_calibrated[i] = QTc_DNN_test[i]
    END IF
    FOR j in [3,2,1]
        IF (QTc_DNN_test[i] + biases[j]) ≥ thresholds[j]:
            QTc_calibrated[j] = min(500, QTc_DNN_test[i] + biases[j])

        END IF
    END FOR
END FOR

RETURN QTc_calibrated
```
